# Association of short-term exposure to sulfur dioxide and hospitalization for ischemic and hemorrhagic stroke in Guangzhou, China

**DOI:** 10.1186/s12889-020-8354-0

**Published:** 2020-02-21

**Authors:** Shuqun Shen, Xing Li, Chao Yuan, Qin Huang, Dongyang Liu, Shuoyi Ma, Jialiang Hui, Ruiyu Liu, Tongwei Wu, Qing Chen

**Affiliations:** 1 School of Public Health, Southern Medical University, 1838#Guangzhou North Avenue, Guangzhou, Guangdong 510515 People’s Republic of China; 2 Guangdong Provincial Institute of Public Health, Guangdong Provincial Center for Disease Control and Prevention, Guangzhou, Guangdong 510515 People’s Republic of China; 3 Department of Neurology, Nanfang Hospital, Southern Medical University, 1838#Guangzhou North Avenue, Guangzhou, Guangdong 510515 People’s Republic of China; 4 Department of Cardiovascular Surgery & Center for Basic Medical Research, TEDA International Cardiovascular Hospital, Chinese Academy of Medical Sciences, & Nankai University, Tianjin, 300457 China; 5 Department of Rheumatology, Nanfang Hospital, Southern Medical University, 1838#Guangzhou North Avenue, Guangzhou, Guangdong 510515 People’s Republic of China; 6 Department of Medicine, Brigham and Women’s Hospital and Harvard Medical School, Boston, MA 02115 USA; 7 Department of Pain Management, The State Key Clinical Specialty for Pain Medicine, The Second Affiliated hospital, Guangzhou Medical University, Guangzhou, Guangdong 510260 People’s Republic of China; 8 Department of Interventional Radiology, Guangzhou First People’s Hospital, School of Medicine, South China University of Technology, No.1 Panfu Road, Guangzhou, 510180 Guangdong People’s Republic of China; 9 First Clinical Medicine College, Southern Medical University, 1838#Guangzhou North Avenue, Guangzhou, Guangdong 510515 People’s Republic of China

**Keywords:** Sulfur dioxide, Ischemic stroke, Hemorrhagic stroke, Hospital admission, China

## Abstract

**Background:**

In developing countries, ambient sulfur dioxide (SO_2_) is a serious air pollutant concern, but there is no enough and consistent epidemiological evidence about its health effects on stroke hospitalization.

**Methods:**

We collected the daily air pollution data, meteorological data and number of daily hospital admissions for ischemic and hemorrhagic stroke, in Guangzhou from January 1st 2009 to December 31st 2014. Then we applied generalized additive model with a quasi-Poisson link to assess the relationship between short-term SO_2_ exposure and the total number of hospital admissions for ischemic and hemorrhagic stroke. In addition, we evaluated the effect of ambient SO_2_ by age (< 65 years and ≥ 65 years).

**Results:**

During the study period, a 24-h mean concentration of ambient SO_2_ of 27.82 μg/m^3^, a total of 58,473 ischemic stroke and 9167 hemorrhagic stroke hospital admissions hospital were recorded. Ambient SO_2_ was found to increase the risk for both ischemic and hemorrhagic stroke hospital admission in single pollutant model. The maximum value of percentage changes for ischemic and hemorrhagic stroke occurred in lag 0 day and lag 1 day, per 10 μg/m^3^ increase in SO_2_ concentrations was corresponded to a 1.27% (95% confidence interval (CI), 0.42–2.12%) and 1.55% (95%CI, 0.02–3.11%) increased risk, respectively. The association between SO_2_ and ischemic stroke hospitalization was robust to two pollutant model, but for hemorrhagic stroke it’s partially weakened after adjusting for co-pollutants. The effect of ambient SO_2_ on ischemic stroke appeared to be greater for people < 65 years old, but null effect on hemorrhagic stroke was identified for both age groups.

**Conclusions:**

We found short-term exposure to ambient SO_2_ may significantly increase the risks of hospitalization for ischemic stroke. The findings may contribute to a better understanding of the health effects of low-levels of SO_2_.

## Background

Stroke is the second leading cause of death in the world [1]. According to Global Burden of Disease (GBD) Study 2016, the highest age-standardized incidences of stroke were observed in east Asia, especially China (354 [95% UI 331–378] per 100,000 person-years) [2]. Ischemic stroke and hemorrhagic stroke account for approximately 70 and 28% of all stroke cases, respectively [3]. Moreover, the incidence of stroke continues to rise in China in recent years [3]. Therefore, identification of modifiable risk factors for stroke has significant public health implications.

Hypertension, diabetes mellitus and dyslipidemia are major risk factors for cardiovascular diseases including stroke [4, 5]. The high prevalence of these risk factors is a major burden to most economies around the world. Ambient air pollution is also a well-known risk factor for stroke morbidity [6], but the majority of evidence are focused on particulate matter (PM) [7, 8]. Sulfur dioxide (SO_2_) is a non-flammable, colorless gas with a strong pungent odor at room temperature, which is primarily released from the combustion of sulfur containing fossil fuels at power plants (73%) and other industry facilities (20%) [9]. As a strong respiratory irritant and bronchoconstrictor, SO_2_ could induce systemic inflammation and oxidative stress [10], which might take a role in stroke. Short-term exposure to ambient SO_2_ was associated with increased risk of stroke morbidity in a meta-analysis mainly based on developed countries [6]. However, in developing countries like China, the evidence is limited and very few studies [11] distinguished between ischemic and hemorrhagic stroke. Most studies only focused on ischemic stroke but the results have not been conclusive yet, with some finding evidence of a positive association [12] but not in other studies [13–16].

Guangzhou is one of the most densely populated cities in China. The industrial production and traffic burden make SO_2_ one of the main atmospheric pollution sources in Guangzhou [16], which is severer than those in developed countries [17]. Moreover, the stroke mortality in Guangzhou was relatively higher compared to other cities of China [18].

Therefore, we conducted a time-series study to examine the short-term association between ambient SO_2_ and hospital admissions of ischemic and hemorrhage stroke in Guangzhou, respectively.

## Method

### Study setting

Guangzhou is located in the South China, which has a typical subtropical humid-monsoon climate with an average annual temperature of 22 °C and an average rainfall of 1500–2000 mm [19]. There are 12.7 million people lived in Guangzhou in 2010 [20]. The city is one of the top-ranking commercial and manufacturing regions in China. As the economy develops in Guangzhou, it also introduces severe air pollution problems which might lead to risks to human health [21].

### Data sources

We obtained the air quality monitoring data from the public sharing system of Guangzhou Environmental Monitoring Center (http://210.72.1.33:8023/gzaqi_new/RealTimeDate.html), which includes data of all the 11 national air quality monitor stations in Guangzhou. The data of SO_2_, nitrogen dioxide (NO_2_) and suspended particulates smaller than 10 μm in aerodynamic diameter (PM_10_) covered the period from January 1, 2009 to December 31, 2014. But for suspended particulates smaller than 2.5 μm in aerodynamic diameter (PM_2.5_) and ozone (O_3_), the data was limited to the period of March 8, 2012 to December 31, 2014, because Guangzhou Environmental Monitoring Center started the surveillance of PM_2.5_ and O_3_ from March 8, 2012. All data from the monitoring stations were averaged in the present study. We used daily average concentration for SO_2_, NO_2_, PM_10_, PM_2.5_, and daily maximum 8-h average (O_3_-8h max) for O_3_.

Daily meteorological data (daily mean temperature and relative humidity) from January 1, 2009 to December 31, 2014 were obtained from the China Meteorological Data Sharing Service System. All meteorological stations were averaged in the analysis.

The data on air pollution and meteorology follow the quality control programs mandated by the Chinese government. These types of data have been widely used to explore their effects on morbidity and mortality in Guangzhou [22–24].

Daily hospital admissions data were obtained from eight hospitals in Guangzhou during January 1, 2009 to December 31, 2014. If there were recurrent strokes occurring within 28 days of a previous admission, then it was treated as a single stroke event [25]. All the cases were coded under the World Health Organization’s International Classification of Diseases, the 10th version (ICD-10). Ischemic and hemorrhagic stroke hospital admissions were identified as I63 and I61, respectively.

This study does not involve experimental animals or individual information on human subjects. Ethical approval was not required for this study. Research projects that do not involve human participants, their data or tissue do not require ethical review by the ethics committee in Southern Medical University.

### Statistical analysis

To assess the relationship between short-term exposure of SO_2_ and stroke hospital admissions, we applied a generalized additive model (GAM) with Poisson link in this study [26]. In accordance with previous studies, we used 7 degrees of freedom (df) per year for the smooth function of calendar time to control for fluctuations in hospitalization on long time trend and seasonality [27], 6 df for the current day’s temperature, and 3 df for relative humidity [28]. Day of the week (DOW) was also included as a dummy variable.

We first used the single pollutant model to examine the independent association between SO_2_ and ischemic/hemorrhagic stroke risk. Then two pollutant model including another pollutant was built to test if there are any potential cofounding effects of other pollutant (e.g. NO_2_) on the relationship between SO_2_ and stroke.

These models can be generically represented as:

Single pollutant model:

Log [E (Y_t_)] = α + β_i_X_i_ + s (temperature, df = 6) + s (humidity, df = 3) + DOW + s (time, df = 7/year).

Two pollutants model:

Log [E (Y_t_)] = α + ∑β_i_X_i_ + s (temperature, df = 6) + s (humidity, df = 3) + DOW + s (time, df = 7/year).

where E (Y_t_) stands for the expected number of stroke hospital admissions on day t. α represent the intercept. X is the concentrations of pollutant (SO_2_, PM_10_, PM_2.5_, NO_2_ and O_3_). β_i_ stands for the coefficient for X_i_. s() represented a smoother based on penalized smoothing splines, which captures the nonlinear relationships of the covariates of time trend, temperature and humidity with daily hospitalization. df represents the degree of freedom. Daily mean temperature and relative humidity were used in all models to control for confounding. Time is the time to adjust for long-term trend and seasonality.

To explore the potential modification, we examined outcomes by stratification of age (≥65 years and < 65 years). We also conducted sensitivity analyses to check the robustness of results by changing the degrees of freedom in the smooth function of time trend (7–9 per year). *P* value < 0.05 was considered as statistically significant. All the analyses were performed with R software (R Development Core Team, 2015).

## Results

Table 1 presented the stroke, meteorological and air pollution data in Guangzhou during January 1, 2009 to December 31, 2014. Overall, 58,473 ischemic stroke and 9167 hemorrhagic stroke hospital admissions were recorded, the distribution of age is presented in Supplementary Table 1. The daily average temperature was 21.99 °C while daily relative humidity was 76.30%. The average concentrations of air pollutants including SO_2_, PM_10_, PM_2.5_, NO_2_ and O_3_ were 27.82 μg/m^3^, 72.93 μg/m^3^, 46.07 μg/m^3^, 54.33 μg/m^3^ and 68.69 μg/m^3^, respectively.

**Table 1:** Daily mortality, weather and air pollution data in Guangzhou (2009–2014)

	Mean (SD)	Min	P_25_	P_50_	P_75_	Max
Daily stroke hospital admissions						
Ischemic stroke	27 (18)	1	14	21	35	101
Hemorrhagic stroke	4 (3)	0	2	4	6	15
Weather						
Temperature (°C)	21.99 (6.37)	4.80	17.40	23.40	27.30	32.30
Humidity (%)	76.30 (12.48)	27.00	69.00	79.00	85.00	100.00
Air pollutant						
SO_2_ (μg/m^3^)	27.82 (18.85)	2.00	14.42	22.21	36.00	116.00
NO_2_ (μg/m^3^)	54.33 (23.90)	0.00	37.87	49.32	65.93	24.100
PM_10_ (μg/m^3^)	72.93 (42.15)	3.00	44.00	64.00	94.41	417.00
PM_2.5_ (μg/m^3^)^a^	46.07 (24.77)	6.90	28.78	43.40	62.86	156.35
O_3_ (μg/m^3^)^a^	68.69 (25.50)	7.00	51.27	85.80	127.91	326.55

Abbreviations: *SO*_*2*_ sulfur dioxide; *NO*_*2*_ nitrogen dioxide; *O*_*3*_ ozone; *PM*_*10*_ particulate matter with aerodynamic diameter less than 10 μm; *PM*_*2.5*_ particulate matter with aerodynamic diameter less than 2.5 μm; P_25,_ P_50_, P_75_, the 25th, 50th and 75th percentile

^a^, data were in the period of March 8, 2012 to December 31, 2014

The correlation coefficients between SO_2_ and other pollutants varied from 0.31 to 0.48. And SO_2_ is negatively correlated with humidity, which is opposite to temperature. (Supplementary Table 2).

For ischemic stroke, we observed the maximum effect of SO_2_ at lag0 day (Table 2). Every 10 μg/m^3^ increase of SO_2_ was associated with 1.27% (95%CI, 0.42–2.12%) increase of ischemic stroke (Table 2). In all the two-pollutant models, the effects of SO_2_ remained in line with that of single pollutant model (Table 4). When looking into the different age groups, a 10 μg/m^3^ increase of SO_2_ concentrations on lag 0 day represented 1.39% (95%CI, 0.35–2.45%) and 1.21% (95%CI, 0.02–2.42%) increment for ischemic stroke hospital admissions in < 65 and ≥ 65 years old group, respectively (Table 3). And we found SO_2_ still increased the risk of hemorrhagic stroke hospital admission with the adjustment of NO_2_ and O_3_ for < 65 years (Table 4). But null association existed in two pollutant model for ≥65 years (Table 4).

**Table 2:** The percent change of stroke risk associated with 10 μg/m^3^ and per IQR increase of SO_2_ in different lag days in single pollutant model

Lag Day		Ischemic stroke	Hemorrhagic stroke
Lag 0	per 10 μg/m^3^	**1.27 (0.42–2.12)**	1.48 (− 0.07 - 3.05)
	per IQR	**2.75 (0.90–4.63)**	3.22 (− 0.15 - 6.69)
Lag 1	per 10 μg/m^3^	**0.93 (0.09–1.78)**	**1.55 (0.02–3.11)**
	per IQR	**2.02 (0.19–3.88)**	**3.38 (0.04–6.82)**
Lag 2	per 10 μg/m^3^	0.37 (− 0.45 - 1.21)	**1.52 (0.02–3.04)**
	per IQR	0.81 (− 0.98 - 2.62)	**3.30 (0.04–6.67)**
Lag 3	per 10 μg/m^3^	0.48 (− 0.34 - 1.30)	0.92 (− 0.55 - 2.41)
	per IQR	1.04 (− 0.73 - 2.83)	1.99 (− 1.19 - 5.27)

Abbreviations: *IQR* interquartile increase; *NO*_*2*_ nitrogen dioxide; *O*_*3*_ ozone; *PM*_*10*_ particulate matter with aerodynamic diameter less than 10 μm; *PM*_*2.5*_ particulate matter with aerodynamic diameter less than 2.5 μm; *SO*_*2*_ sulfur dioxide

**Table 3:** The percent change of stroke risk associated with 10 μg/m^3^ and per IQR increase of SO_2_ in different lag days and age group (single pollutant model)

Lag Day		Ischemic stroke		Hemorrhagic stroke	
		< 65 years	≥65 years	< 65 years	≥65 years
Lag 0	per 10 μg/m^3^	**1.39 (0.35–2.45)**	**1.21 (0.02–2.42)**	1.55 (− 0.14 - 3.26)	1.04 (− 2.04 - 4.23)
	per IQR	**3.03 (0.75–5.36)**	**2.64 (0.05–5.30)**	3.37 (− 0.29 - 7.18)	2.27 (−4.36 - 9.35)
Lag 1	per 10 μg/m^3^	**1.31 (0.27–2.35)**	0.68 (− 0.50 - 1.88)	1.47 (− 0.18 - 3.16)	0.25 (− 2.79 - 3.40)
	per IQR	**2.84 (0.59–5.14)**	1.48 (− 1.08 - 4.10)	3.21 (− 0.39 - 6.93)	0.55 (− 5.93 - 7.48)
Lag 2	per 10 μg/m^3^	0.80 (− 0.22 - 1.82)	0.03 (− 1.13 - 1.20)	0.65 (− 0.97 - 2.30)	2.31 (− 0.69 - 5.40)
	per IQR	1.73 (− 0.47 - 3.98)	0.06 (− 2.43 - 2.61)	1.41 (− 2.09 - 5.03)	5.06 (− 1.48 - 12.03)
Lag 3	per 10 μg/m^3^	0.82 (− 0.19 - 1.83)	0.24 (− 0.91 - 1.40)	1.18 (− 0.42 - 2.81)	− 0.57 (− 3.49 - 2.44)
	per IQR	1.77 (− 0.4 - 3.99)	0.51 (− 1.95 - 3.04)	2.57 (− 0.90 - 6.16)	− 1.23 (− 7.39 - 5.33)

Abbreviations: *IQR* interquartile increase; *NO*_*2*_ nitrogen dioxide; *O*_*3*_ ozone; *PM*_*10*_ particulate matter with aerodynamic diameter less than 10 μm; *PM*_*2.5*_ particulate matter with aerodynamic diameter less than 2.5 μm; *SO*_*2*_ sulfur dioxide

**Table 4:** Percentage increase of stroke risk associated with per 10 μg/m^3^ and per IQR increase of SO_2_ under single and two-pollutant models

Age group	Pollutant	Model type	Ischemic stroke^a^		Hemorrhagic stroke#	
			per 10 μg/m^3^ (95%CI)	per IQR (95%CI)	per 10 μg/m^3^ (95%CI)	per IQR (95%CI)
All age	SO_2_	Single pollutant	**1.27 (0.42–2.12)**	**2.75 (0.90–4.63)**	**1.55 (0.02–3.11)**	**3.38 (0.04–6.82)**
		Two-pollutant				
		**SO**_**2**_ + NO_2_	**1.34 (0.31–2.37)**	**2.90 (0.66–5.20)**	1.25 (− 0.48 - 3.01)	2.72 (− 1.04 - 6.62)
		**SO**_**2**_ + PM_10_	**1.21 (0.18–2.26)**	**2.64 (0.39–4.94)**	0.89 (− 0.89 - 2.7)	1.93 (− 1.91 - 5.91)
		**SO**_**2**_ + PM_2.5_	**2.25 (0.06–4.48)**	**4.91 (0.12–9.93)**	2.71 (− 1.39 - 6.98)	5.94 (− 2.98 - 15.68)
		**SO**_**2**_ + O_3_	**2.68 (0.75–4.65)**	**5.88 (1.63–10.3)**	**4.32 (0.63–8.15)**	**9.56 (1.36–18.43)**
< 65 years	SO_2_	Single pollutant	**1.39 (0.35–2.45)**	**3.03 (0.75–5.36)**	1.47 (− 0.18 - 3.16)	3.21 (− 0.39 - 6.93)
		Two-pollutant				
		**SO**_**2**_ + NO_2_	**1.59 (0.36–2.83)**	**3.46 (0.78–6.21)**	1.07 (− 0.8 - 2.97)	2.32 (− 1.71 - 6.51)
		**SO**_**2**_ + PM_10_	1.22 (− 0.02 - 2.47)	2.64 (− 0.04 - 5.40)	1.37 (− 0.55 - 3.33)	2.98 (− 1.18 - 7.32)
		**SO**_**2**_ + PM_2.5_	2.23 (− 0.39 - 4.93)	4.88 (− 0.85 - 10.94)	3.94 (− 0.58 - 8.68)	8.71 (− 1.25 - 19.67)
		**SO**_**2**_ + O_3_	**3.70 (1.36–6.09)**	**8.15 (2.95–13.60)**	**4.45 (0.38–8.69)**	**9.85 (0.82–19.69)**
≥65 years	SO_2_	Single pollutant	**1.21 (0.02–2.42)**	**2.64 (0.05–5.30)**	0.25 (− 2.79 - 3.40)	0.55 (− 5.93 - 7.48)
		Two-pollutant				
		**SO**_**2**_ + NO_2_	1.09 (− 0.32 - 2.51)	2.36 (− 0.69 - 5.51)	0.67 (− 2.79 - 4.26)	1.45 (− 5.93 - 9.42)
		**SO**_**2**_ + PM_10_	1.19 (− 0.23 - 2.63)	2.58 (− 0.5 - 5.75)	− 1.21 (− 4.71 - 2.41)	−2.6 (− 9.89 - 5.28)
		**SO**_**2**_ + PM_2.5_	2.19 (− 0.49 - 4.94)	4.78 (− 1.06 - 10.96)	− 0.96 (− 8.36 - 7.03)	− 2.06 (− 17.17 - 15.8)
		**SO**_**2**_ + O_3_	1.90 (− 0.45 - 4.31)	4.14 (− 0.98 - 9.52)	1.62 (− 5.14 - 8.85)	3.52 (− 10.76 - 20.08)

Abbreviations: *IQR* interquartile increase; *NO*_*2*_ nitrogen dioxide; *O*_*3*_ ozone; *PM*_*10*_ particulate matter with aerodynamic diameter less than 10 μm; *PM*_*2.5*_ particulate matter with aerodynamic diameter less than 2.5 μm; *SO*_*2*_ sulfur dioxide

Ischemic stroke^a^: models were based on lag 0 (maximum effect day in single model). Hemorrhagic stroke#: models were based on lag 1 (maximum effect day in single model)

For hemorrhagic stroke, the effect of SO_2_ reached the maximum value on lag1 day (Table 2), with percentage change of 1.55% (95%CI, 0.02–3.11%) per 10 μg/m^3^. But neither of the effect is significant in < 65 and ≥ 65 years old group (Table 3). The effect of SO_2_ lost statistical significance after including NO_2_, PM_10_ and PM_2.5_ but it’s still significant when O_3_ was included (Table 4).

Sensitivity analyses were performed to check the robustness of results in terms of the degrees of freedom in the smooth function of time trend, which was similar to that in the main model (Supplementary Table 3).

## Discussion

This is one of the few studies specified not only association between SO_2_ and ischemic stroke but also hemorrhagic stroke hospital admissions in developing countries so far.

Our study suggested that ambient SO_2_ was significantly associated with increased hospital admissions for ischemic stroke. A 10 μg/m^3^ increase of the same day/lag 1 day SO_2_ was associated with increments of 1.27% (0.42–2.12%) and 0.93% (0.09–1.78%) ischemic stroke hospital admissions risk, respectively. The associations were robust to the adjustment of all the other air pollutants. For example, the most informative pollutant of traffic emissions NO_2_. The association were comparable to previously studies [6, 11, 12, 16].

However, the associations of SO_2_ and hemorrhagic stroke were more variable and imprecise. We observed significant associations between lag 1/lag 2 days SO_2_ exposures and hemorrhagic stroke hospitalization risk, with percentage change of 1.55% (0.02–3.11%) and 1.52% (0.02–3.04%), respectively. But when adjusted for other air pollutants in our models (except O_3_), the magnitude of risk due to SO_2_ exposure turned out to be statistically insignificant. Previous evidences on SO_2_-hemorrhagic stroke association are scarce and inconsistent [11, 29]. The discrepant results on hemorrhagic stroke may be explained by differences in average pollutant levels, meteorological patterns and population characteristics between studies [30]. For example, in the study of Liu et al. [11], SO_2_ concentration level was 39.6 ± 41.2 μg/m^3^ with remarkably higher variation than that in Guangzhou (21.99 ± 6.37 μg/m^3^) in our study. The exposure levels and exposure patterns might partly explain the difference.

The identification of potentially vulnerable subpopulation has significant implications for public health [12], therefore we stratified the stroke cases into < 65 and ≥ 65 years groups. For ischemic stroke, we found that the effect estimates of SO_2_ were higher for the < 65 than that in the ≥65 age group, which is the opposite of Tian et al. [12]. This may be due to the varied activity patterns for different age groups in different cities. The elderly in Guangzhou tend to stay in door or in the park nearby, while people under 65 years old usually take vehicles or public transportation to work and have more exposure on SO_2_, because the vehicle exhaust emission is one of the main sources of SO_2_. But for hemorrhagic stroke hospitalization, there was no evidence of effect in neither age subgroup in any lag structure. It might be related to the smaller number of daily hospital admissions of hemorrhagic stroke (4 ± 3 cases/day) in this study. The lower incidence of hemorrhagic stroke may lead to larger imprecision in the estimates [6]. In addition, we noted that 70.14% hemorrhagic stroke cases were < 65 years old, while 59.75% ischemic stroke cases were ≥ 65 years old, which was similar to previous observations [11]. But the different age distribution of ischemic and hemorrhagic stroke should not be ignored. It might also contribute to the different relationship between SO_2_ and ischemic and hemorrhagic stroke.

The mechanism behind the adverse stroke effects associated with SO_2_ is biological plausible. The inhalation of SO_2_ could alter heart rate variability, elevate oxidative and enhanced blood coagulation and thrombosis formation [31]. Animal study also showed that SO_2_ elevated the levels of cyclooxygenase-2, interleukin-1b, tumor necrosis factor-a, intercellular adhesion molecule-1 mRNA and protein, which might also play a role in the development of stroke [32]. These pathophysiologic changes may be related to the development and progression of stroke. Nevertheless, the exact biological pathways underlying the health impact of SO_2_ need further investigations.

Although this study found that the magnitude of elevated risk of stroke due to SO_2_ exposures in Guangzhou was relatively small, it should not be ignored. Coal related air pollution, characterized by high level of SO_2_, remains to be an important environmental problem in China, including Guangzhou [10]. The increased risk of ischemic stroke and hemorrhagic stroke due to the exposures of SO_2_ estimated in this work highlights the need for continued vehicle emission control, desulfurization, and promotion of hydropower and nuclear power from the government [33] and vigilance for the health risks of air pollution. There are several limitations of this study. First, averaging air pollution exposures over Guangzhou may introduce exposure measurement error. Secondly, as a retrospective ecological study, personal exposure confounders could not be excluded in the model, and we have no access to individual air pollution exposure data. Further studies based on personal measurements or modeled residential concentrations are warranted to evaluate the health effects of SO_2_, especially at low levels or in the real world. Moreover, the potential misclassifications for the stroke diagnosis should also be taken into consideration. However, all the hospitals included in this study are top-ranked public hospitals with high quality of electronic diseases diagnosis records in Guangzhou. Another limitation of the present study is the lack of consideration of more age groups, as the distribution of ischemic and hemorrhagic stroke might vary across the age (e.g. 65–74, and ≥ 75 years), we recommend future studies to examine the association in subgroups as 65–74, and ≥ 75 years. Due to the limited number of hemorrhagic stroke hospitalizations, studies based on enlarged sample size or in multi-cites should be performed to examine the association in the future. In addition, hypertension, diabetes mellitus and dyslipidemia are important risk factors for stroke [4]. Further studies taking the comorbidity of stroke and hypertension, diabetes mellitus and dyslipidemia into consideration could be helpful to identify effective ways to prevent stroke.

## Conclusions

In summary, we found short-term exposure to ambient SO_2_ may significantly increase the risks of hospitalization for ischemic stroke. The findings may contribute to a better understanding of the health effects of SO_2_.

## Supplementary information

**Additional file 1 Supplementary Table 1.** The distribution of ischemic and hemorrhagic stroke events by age. **Supplementary Table 2.** Correlations between air pollutants and meteorological factors. **Supplementary Table 3.** The percent change of stroke risk associated with 10 μg/m^3^ and per IQR increase of SO_2_ with different df (sensitivity analysis).

## Data Availability

The air pollution data that support the findings of this study are available on request from the corresponding author, Qing Chen. The stroke data are not publicly available due to the privacy restriction. *Competing interests.* The authors declare they have no competing interests.

## Abbreviations

CI: Confidence interval
DOW: Day of the week
GAM: Generalized additive model
GBD: Global Burden of Disease
ICD-10: International Classification of Diseases, the 10th version
O_3_: Ozone
PM: Particulate matter
SO_2_: Sulfur dioxide
