# Supplementary material for: Association of short-term exposure to sulfur dioxide and hospitalization for ischemic and hemorrhagic stroke in Guangzhou, China
Source: BMC Public Health. 2020 Feb 21;20:263. doi: 10.1186/s12889-020-8354-0 (PMC7035656; doi:10.1186/s12889-020-8354-0)
Supplement: Supplementary file 1 — Additional file 1 Supplementary Table 1. The distribution of ischemic and hemorrhagic stroke events by age. Supplementary Table 2. Correlations between air pollutants and meteorological factors. Supplementary Table 3. The percent change of stroke risk associated with 10 μg/m3 and per IQR increase of SO2 with different df (sensitivity analysis). [file 12889_2020_8354_MOESM1_ESM.doc]

**Supplementary material**

**Supplementary Table 1** The distribution of ischemic and hemorrhagic stroke events by age

| Variable | Ischemic stroke | Hemorrhagic stroke |
| --- | --- | --- |
| Total | 39,826 | 4,987 |
| <65 years (%) | 16,029 (40.25) | 3,498 (70.14) |
| ≥65 years (%) | 23,797 (59.75) | 1,489 (29.86) |

**Supplementary Table 2** Correlations between air pollutants and meteorological factors

|  | SO_2_ | NO_2_ | PM_10_ | PM_2.5_ | O_3_ | Humidity | Temperature |
| --- | --- | --- | --- | --- | --- | --- | --- |
| SO_2_ | 1.00 | 0.43 | 0.37 | 0.48 | 0.31 | -0.15 | 0.12 |
| NO_2_ | 0.43 | 1.00 | 0.60 | 0.71 | 0.07 | -0.04 | -0.20 |
| PM_10_ | 0.37 | 0.60 | 1.00 | 0.86 | 0.24 | -0.08 | -0.22 |
| PM_2.5_ | 0.48 | 0.71 | 0.86 | 1.00 | 0.24 | -0.27 | -0.38 |
| O_3_ | 0.31 | 0.07 | 0.24 | 0.24 | 1.00 | -0.38 | -0.38 |
| Humidity | -0.15 | -0.04 | -0.08 | -0.27 | -0.38 | 1.00 | 0.20 |
| Temperature | 0.12 | -0.20 | -0.22 | -0.38 | -0.38 | 0.20 | 1.00 |

Abbreviations: SO_2_, sulfur dioxide; NO_2_, nitrogen dioxide; O_3_, ozone; PM_10_, particulate matter with aerodynamic diameter less than 10 μm; PM_2.5_, particulate matter with aerodynamic diameter less than 2.5 μm.

**Supplementary Table 3** The percent change of stroke risk associated with 10 μg/m^3^ and per IQR increase of SO_2_ with different df (sensitivity analysis)

| Lag Day |  | Ischemic stroke | Hemorrhagic stroke |
| --- | --- | --- | --- |
| Df=6 | per 10μg/m^3^ | 1.17 ( 0.33 - 2.02 ) | 1.58 ( 0.07 - 3.12 ) |
|  | per IQR | 2.54 ( 0.71 - 4.4 ) | 3.45 ( 0.15 - 6.86 ) |
| Df=7 | per 10μg/m^3^ | 1.27 ( 0.42 - 2.12 ) | 1.55 ( 0.02 - 3.11 ) |
|  | per IQR | 2.75 ( 0.90 - 4.63 ) | 3.38 ( 0.04 - 6.82 ) |
| Df=8 | per 10μg/m^3^ | 1.20 ( 0.35 - 2.05 ) | 1.54 ( 0.01 - 3.09 ) |
|  | per IQR | 2.60 ( 0.76 - 4.47 ) | 3.36 ( 0.03 - 6.80 ) |
| Df=9 | per 10μg/m^3^ | 1.41 ( 0.55 - 2.27 ) | 1.66 ( 0.12 - 3.23 ) |
|  | per IQR | 3.06 ( 1.20 - 4.96 ) | 3.62 ( 0.26 - 7.10 ) |
